# RAAS inhibitors in pregnancy, breastfeeding and women of childbearing potential: a review of national and international clinical practice guidelines

**DOI:** 10.1038/s41371-025-01001-z

**Published:** 2025-03-05

**Authors:** Caitlin Greenlees, Christian Delles

**Affiliations:** School of Cardiovascular and Metabolic Health, University of Glasgow, Glasgow, UK

**Keywords:** Health care, Risk factors

## Abstract

Globally prevalent conditions such as hypertension, heart failure, ischaemic heart disease (IHD) and chronic kidney disease (CKD) are frequently and effectively treated with blockers of the renin-angiotensin-aldosterone system (RAAS) as a first line treatment in the UK and worldwide. RAAS blockers are prohibited in pregnancy due to their adverse fetal effects. We reviewed clinical guidelines from the National Institute of Health and Care Excellence (NICE) on the management of cardiovascular and kidney disease with RAAS blockers in pregnancy, with other UK, European and American guidance as comparators. Whilst guidelines agree on the strict avoidance of RAAS blockers in pregnancy, nuanced considerations regarding prescription in women of childbearing potential, contraception, timing of RAAS blocker withdrawal and breastfeeding are not consistently addressed in clinical guidelines. We call for consistent wording and more explicit advice on RAAS blocker prescription in women of childbearing potential, in pregnancy and in the postpartum period in future iterations of clinical guidelines.

## Introduction

Cardiovascular and renal diseases pose significant health challenges globally, including conditions such as hypertension, heart failure, ischaemic heart disease (IHD), and chronic kidney disease (CKD). These conditions require careful management throughout life, but particularly during pregnancy to preserve the health of both the mother and the fetus [1].

Blockers of the renin-angiotensin-aldosterone system (RAAS), consisting of direct renin inhibitors (DRIs), angiotensin-converting enzyme inhibitors (ACEIs), angiotensin II receptor blockers (ARBs) and mineralocorticoid receptor antagonists (MRAs) have been integral in the treatment of cardiovascular and renal conditions [2]. They are often used as a first-line treatment for these conditions, especially in younger individuals, but are contraindicated during pregnancy due to their adverse effect on fetal development.

The fetal RAAS plays a crucial role in blood pressure and electrolyte regulation, blood vessel growth, lung and kidney development. Blocking the RAAS causes hypoperfusion of the fetal kidney, which in turn causes anuria, and therefore results in an insufficient amniotic fluid volume in the amniotic sac. Treatment with ACEIs and ARBs can cause renal tubular dysplasia, lung hypoplasia, problems with skull formation and refractory hypotension in the fetus as a result [3]. Fetal malformations resulting from RAAS blockade are linked to inhibition of RAAS activity during the later stages of pregnancy due to differing expression of fetal AT1 and AT2 receptors throughout gestation [4].

Clinical guidelines advise against the use of these drugs for individuals planning a pregnancy or during pregnancy. However, the degree to which the nuanced issue of prescription to women of childbearing potential, planning a pregnancy and pregnancy itself is addressed and justified by current clinical recommendations for the use of RAAS blockers deserves investigation.

This investigation is particularly relevant in instances where a woman may be pregnant, and (due to delayed awareness or a lack of knowledge of the risks) continues to take RAAS blockers and compromise the health of the fetus. Some clinicians may argue that prescribing RAAS blockers to any woman of childbearing age is therefore not appropriate. Inconsistencies also extend to advice on contraception for those who are on RAAS blockers and breastfeeding. For this report we reviewed examples of existing clinical guidelines (Provided in Supplementary Table 1).

## Pregnancy

Guidance overall varies in the inclusion or non-inclusion of pregnancy (Table 1). Hypertension guidance from the National Institutes of Health and Care Excellence (NICE) [5, 6], European Society of Hypertension (ESH) [7], European Society of Cardiology (ESC) [8] and the American Heart Association and American College of Cardiology (AHA/ACC) [9] contains considerations of pregnancy and RAAS blockers comprehensively and includes details on prohibition and associated risks. This may be attributed to its high prevalence and broader impact on maternal and fetal health as a risk factor for other conditions. Specific guidance on the treatment of hypertension and other cardiovascular diseases is available from NICE [6] and the ESC [10] whilst the ESH published a position paper with detailed information but without having guideline status [11]. Details on hypertension guidance can be found in Supplementary Table 2.

**Table 1:** An overview of notable aspects of pregnancy in relation to renin-angiotensin-aldosterone system (RAAS) blockade covered by published clinical guidelines.

		Hypertension	Heart Failure	IHD	CKD
NICE [5, 6, 12, 15, 18]	Prohibition of RAAS blockers	Y	N	N	N
	Discontinuation timing	Y	N	N	N
	Childbearing potential	Y	N	N	N
	Breastfeeding/lactation	Y	N	N	N
	Replacements	Y	Y	N	N
ESH [7, 11]	Prohibition of RAAS blockers	Y			
	Discontinuation timing	Y			
	Childbearing potential	Y			
	Breastfeeding/lactation	Y			
	Replacements	Y			
ESC [8, 10]	Prohibition of RAAS blockers	Y	Y	N	
	Discontinuation timing	Y	Y	N	
	Childbearing potential	Y	N	N	
	Breastfeeding/lactation	Y	Y	N	
	Replacements	Y	Y	N	
AHA/ACC [9, 14, 17]	Prohibition of RAAS blockers	Y	Y	Y	
	Discontinuation timing	N	N	N	
	Childbearing potential	N	N	N	
	Breastfeeding/lactation	N	Y	Y	
	Replacements	Y	Y	Y	
The Renal Association (UK) [19]	Prohibition of RAAS blockers				Y
	Discontinuation timing				N
	Childbearing potential				Y
	Breastfeeding/lactation				Y
	Replacements				Y
KDIGO [20]	Prohibition of RAAS blockers				Y
	Discontinuation timing				Y
	Childbearing potential				Y
	Breastfeeding/lactation				Y
	Replacements				Y

Hypertension, heart failure, ischaemic heart disease (*IHD*) and chronic kidney disease (*CKD*) are included. Y = Yes / Guidance is included; N = No / Guidance is not included; Blank = Guidelines not available.

*IHD* ischaemic heart disease, *CKD* chronic kidney disease, *RAAS* renin-angiotensin-aldosterone system, *NICE* national institute of health and care excellence, *ESH* european society of hypertension, *ESC* european society of cardiology, *CVD* cardiovascular disease, *AHA/ACC* american heart association/american college of cardiology, *KDIGO* kidney disease: improving global outcomes.

More variability in recommendations can be observed across other cardiovascular and renal conditions. For example, the NICE 2018 guidelines for heart failure management [12] caution against pregnancy for those of childbearing potential with heart failure. The 2018 NICE guidance has no mention of specific considerations regarding RAAS blockers above the level of pregnancy contraindication. The 2021 ESC heart failure guidelines [13] and the 2018 ESC cardiovascular disease in pregnancy guidelines [10] detail specifically mention pre-pregnancy management of existing medications. This contrasts the AHA/ACC 2022 heart failure guidance [14], which details the risks associated with RAAS blockers and does not mention the avoidance of pregnancy (Supplementary Table 3). The comparatively limited guidance for heart failure might indicate the complexity of managing this condition in pregnancy, especially in severe cases, where the focus revolves around advising against pregnancy. Similarly, while NICE [15] and ESC [16] guidelines lack any pregnancy related IHD guidance, the AHA/ACC [17] guidance offer comprehensive recommendations, strongly advising against RAAS blockers during pregnancy, detailing potential fetal harm and recommending multidisciplinary preconception care in women with IHD (Supplementary Table 4). This same picture can be seen in NICE guidelines for the management of CKD [18], which do not include pregnancy considerations whereas guidance from The Renal Association 2019 [19] and Kidney Disease: Improving Global Outcomes (KDIGO) 2024 [19] highlights the teratogenic profile of RAAS blockers and includes comprehensive and personalised pregnancy management recommendations. Moreover, The Renal Association 2019 [20] guidelines have detailed recommendations for pre-pregnancy counselling and medication management in women of childbearing potential (Supplementary Table 5). IHD is the number one cause of fatalities in women globally [21]. The lack of detailed guidance or consensus on managing IHD during pregnancy poses a challenge.

There are conditions that may present compelling indications for RAAS-blocking agents, for example, mineralocorticoid receptor antagonists (MRAs) in primary aldosteronism secondary to adenoma, hyperplasia or familial types. Women of childbearing potential living with these conditions would be at risk of adverse effects of MRAs in pregnancy, but may also benefit from pregnancy due to progesterone physiologically acting as MRA [22, 23], emphasising the importance of individualised decision-making [24].

## Childbearing potential and contraception

Guidelines concerning women of childbearing potential and RAAS blockers vary in the recommendations. While NICE [5] recommends treating hypertension based on general adult guidelines, the guidance lacks explicit contraception or counselling advice. Comparatively, the KDIGO 2024 CKD guidance [20] specifically refers to childbearing potential and advocates for a multidisciplinary, individualised approach where contraception and preconception counselling are considered. The Renal Association [19] also offers detailed directives for women of childbearing potential, with guidance recommending a discussion of a personalised plan for discontinuation, governed by the likelihood of pregnancy and the level need for the medication. The ESH hypertension and cardiovascular disease in pregnancy position paper [11] advises caution in prescribing ACEIs and ARBs to women “without reliable contraception” and includes directives for patients who have unknowingly been exposed to these medications in pregnancy. Conversely, the ACC/AHA hypertension guideline [9] has no specific statement on childbearing potential other than strictly prohibiting RAAS blockers during pregnancy. The absence of consideration for women of childbearing potential in the NICE and ACC/AHA guidelines may still signal a distinction in treatment between pregnant and non-pregnant women but allows a broader scope of discretion for clinicians and may contribute to avoidable sex-based health inequalities. Zhao et al. 2020 [25] found significant disparities in medication prescription between sexes, with women being less likely to receive ACEIs than men, highlighting the need for documented counselling and contraception advice.

## Discontinuation of RAAS blockers upon notification of pregnancy and suitable replacements

The 2019 NICE guidance for hypertension [5] advises discontinuing RAAS blockers within “2 working days of notification of pregnancy”. Similarly, The Renal Association [19] recommends the same timeframe of two days of notification of pregnancy for women with CKD. In contrast, the 2023 ESH hypertension guidance [7] suggests discontinuation of RAAS blockers in the first trimester (Supplementary Table 2). The 2021 and 2018 heart failure guidance from ESC [13] advises that RAAS blockers should be stopped prior to conception and includes recommendations for situations where the medication may have been unknowingly taken during the first trimester (Supplementary Table 3). KDIGO 2024 guidelines [20] recommend discontinuation of ACEis and ARBs during pregnancy with no specific timeframe indicated. A 2010 UK survey found that 86% of women sought prenatal care by gestational week 12, indicating a potential delay in care for 14% of participants until after the first trimester [26].

Research suggests that there may be potential risks even in early pregnancy stages [3], aligning with NICE’s specific timeline for discontinuation. In terms of an unplanned pregnancy, the lack of clear directives from NICE in other condition-specific guidelines, specifically for heart failure, IHD and CKD may lead to delays in appropriate care (Supplementary Tables 3–5). Additionally, the absence of precise timelines in these guidelines may cause variations in clinical decision-making.

The recommended replacement medication for women who are pregnant or planning a pregnancy shows some uniformity across guidelines and conditions. The NICE hypertension guidelines [5, 6] currently recommend labetalol, nifedipine and methyldopa as equally suitable replacements for RAAS blocker medication during pregnancy. This recommendation from NICE is similar to the corresponding European and US hypertension guidelines. However, the European hypertension guidelines expand on this by evaluating the safety evidence for each medication, with labetalol and nifedipine being reported to have the most safety evidence.

The ongoing Giant PANDA trial [27] aims to further inform treatment by comparing the effect of labetalol and nifedipine for managing high blood pressure during pregnancy. This currently recruiting trial may have the potential to shape future NICE guidance on this topic.

## Lactation and breastfeeding

Recommendations on RAAS blocker prescription during breastfeeding vary among guidelines. While AHA/ACC hypertension guidelines [9] and KDIGO CKD guidelines [20] offer comprehensive coverage, NICE and ESH provide some less detailed guidance on breastfeeding in the hypertension guidelines. NICE guidance states that ACEIs and ARBs are not advised but not absolutely prohibited during breastfeeding but does not specify specific drug names [5]. The 2023 hypertension guidance from the ESH states that “ACEis are compatible with breastfeeding… ARBs are not currently recommended in lactating women because of limited safety evidence” [7] (Supplementary Table 2). The recent 2024 ESC hypertension guidelines also include the specific medication names (Benazepril, captopril, enalapril, quinapril) [8]. Furthermore, both the AHA/ACC guidance for heart failure [14] and IHD guidelines [17] state that enalapril, benazepril and captopril are safe during breastfeeding and lactation in contrast with the NICE guidance (Supplementary Tables 3 and 4). This guidance from the AHA/ACC is reinforced by research showing that drug levels of these ACEIs in breastmilk are low enough that it does not pose any danger on the infant [28, 29]. Similarly, the KDIGO 2024 CKD guidelines [20] advocate for a multidisciplinary care approach where medication charts are reviewed throughout lactation as well as throughout pregnancy. This comparison highlights the inconsistency between the more detailed international guidance and the NICE guidelines. Additionally, specifically addressing prematurity in the guidance may be beneficial, as premature infants are more sensitive to RAAS modulations and build-up of drugs [30].

## Summary

There are disparities in the depth and extent of national and international guidance concerning the use of RAAS blockers during pregnancy and breastfeeding. This presents a challenge, as it demonstrates that there is a lack of standardised and comprehensive guidance on this topic across international levels and suggests that there is a requirement for more explicit directives in the clinical guidelines aimed at pregnant women and women of childbearing potential.

The position statement by the ESH Working Group on Hypertension in Women reinforces the importance of multidisciplinary approaches to hypertensive disorders in pregnancy and attributes the lack of standardised disease management to the multifaceted nature of hypertensive disorders in pregnancy and the scarcity of well-designed clinical trials in the field [11]. Building on this, we recommend that in future iterations and updates of clinical guidelines for the management of cardiovascular and renal conditions that explicit and detailed directives regarding the use of RAAS blockers for both pregnant women and women of childbearing potential are provided, with an emphasis on the importance of appropriate contraception and preconception counselling. Additionally, we recommend that guidance regarding breastfeeding and lactation is clear and includes information on the evidenced safety of specific drugs and further research into the safety of RAAS blockers in breastfeeding. These recommendations will provide healthcare professionals with improved resources to facilitate informed decision-making and patient counselling regarding RAAS blocker treatment during pregnancy and breastfeeding.

## Supplementary information

Supplementary Tables

## Data Availability

All guidelines cited in this paper are available through the links provided in the reference list. Any specific requests can be addressed to the authors.

## References

[1] Buawangpong N, Teekachunhatean S, Koonrungsesomboon N. Adverse pregnancy outcomes associated with first‐trimester exposure to angiotensin‐converting enzyme inhibitors or angiotensin II receptor blockers: a systematic review and meta‐analysis. Pharmacol Res Perspect. 2020;8:e00644. 10.1002/prp2.644. 32815286 10.1002/prp2.644 PMC7438312

[2] Ghazi L, Drawz P. Advances in understanding the renin-angiotensin-aldosterone system (RAAS) in blood pressure control and recent pivotal trials of RAAS blockade in heart failure and diabetic nephropathy. F1000Res. 2017;6:F1000 Faculty Rev-297. 10.12688/f1000research.9692.1. 28413612 10.12688/f1000research.9692.1 PMC5365219

[3] Damato G, Rembouskos G, Cafagna R, Dentico D, Palladino S, Chiarito M, et al. Sustained activation of the renin–angiotensin–aldosterone system after fetal exposure to AT1 blockers: effects on kidney and bone in a preterm newborn. J Obstet Gynaecol Res. 2022;48:3331–5. 10.1111/jog.15427. 36098242 10.1111/jog.15427 PMC10087169

[4] Ray JG, Vermeulen MJ, Koren G. Taking ACE inhibitors during early pregnancy: is it safe? Can Fam Physician. 2007;53:1439–40. 17872870 PMC2234619

[5] National Institute for Health and Care Excellence. Hypertension in adults: diagnosis and management. NICE Guideline. 2019;136. https://www.nice.org.uk/guidance/ng136 [Accessed 4th March 2025].

[6] National Institute for Health and Care Excellence. Hypertension in pregnancy: diagnosis and management. NICE Guideline. 2023;133. https://www.nice.org.uk/guidance/ng133 [Accessed 4th March 2025].

[7] Mancia G, Kreutz R, Brunström M, Burnier M, Grassi G, Januszewicz A, et al. 2023 ESH guidelines for the management of arterial hypertension The Task Force for the management of arterial hypertension of the European Society of Hypertension Endorsed by the European Renal Association (ERA) and the International Society of Hypertension (ISH). J Hypertens. 2023;41:1874–2071. 10.1097/HJH.0000000000003480. 37345492 10.1097/HJH.0000000000003480

[8] McEvoy JW, McCarthy CP, Bruno RM, Brouwers S, Canavan MD, Ceconi C, et al. 2024 ESC guidelines for the management of elevated blood pressure and hypertension. Eur Heart J. 2024;45:3912–4018. 10.1093/eurheartj/ehae178. 39210715 10.1093/eurheartj/ehae178

[9] Whelton PK, Carey RM, Aronow WS, Casey DE, Collins KJ, Dennison Himmelfarb C, et al. Guideline for the prevention, detection, evaluation, and management of high blood pressure in adults. J Am Coll Cardiol. 2018;71:e127–248. 10.1016/j.jacc.2017.11.006. 29146535 10.1016/j.jacc.2017.11.006

[10] Regitz-Zagrosek V, Roos-Hesselink JW, Bauersachs J, Blomström-Lundqvist C, Cífková R, De Bonis M, et al. 2018 ESC guidelines for the management of cardiovascular diseases during pregnancy. Eur Heart J. 2018;39:3165–241. 10.1093/eurheartj/ehy340. 30165544 10.1093/eurheartj/ehy340

[11] Thomopoulos C, Hitij JB, De Backer T, Gkaliagkousi E, Kreutz R, Lopez-Sublet M, et al. Management of hypertensive disorders in pregnancy: a position statement of the European Society of Hypertension Working Group “Hypertension in Women”. J Hypertens. 2024;42:1109–32. 10.1097/HJH.0000000000003739. 38690949 10.1097/HJH.0000000000003739

[12] National Institute for Health and Care Excellence. Chronic heart failure in adults: diagnosis and management. NICE Guideline. 2018;106. https://www.nice.org.uk/guidance/ng106/chapter/Recommendations#treating-heart-failure-with-reduced-ejection-fraction [Accessed 4th March 2025]. 30645061

[13] McDonagh TA, Metra M, Adamo M, Gardner RS, Baumbach A, Böhm M, et al. 2023 Focused Update of the 2021 ESC Guidelines for the diagnosis and treatment of acute and chronic heart failure. Eur Heart J. 2023;44:3627–39. 10.1093/eurheartj/ehad195. 37622666 10.1093/eurheartj/ehad195

[14] Heidenreich PA, Bozkurt B, Aguilar D, Allen LA, Byun JJ, Colvin MM, et al. 2022 AHA/ACC/HFSA guideline for the management of heart failure: A report of the American College of Cardiology/American Heart Association joint committee on clinical practice guidelines. Circulation. 2022;145:e876–e894. 10.1161/CIR.0000000000001062. 35363500 10.1161/CIR.0000000000001062

[15] National Institute for Health and Care Excellence. Stable angina: management. Clinical guideline. 2016. https://www.nice.org.uk/guidance/cg126/resources/stable-angina-management-pdf-35109453262021 [Accessed 4th March 2025].

[16] Knuuti J, Wijns W, Saraste A, Capodanno D, Barbato E, Funck-Brentano C, et al. 2019 ESC guidelines for the diagnosis and management of chronic coronary syndromes. Eur Heart J. 2019;41:407–77. 10.1093/eurheartj/ehz425. 10.1093/eurheartj/ehz425 31504439

[17] Virani SS, Newby LK, Arnold SV, Bittner V, Brewer LC, Demeter SH, et al. 2023 AHA/ACC/ACCP/ASPC/NLA/PCNA guideline for the management of patients with chronic coronary disease: a report of the American Heart Association/American College of Cardiology Joint Committee on Clinical Practice Guidelines. J Am Coll Cardiol. 2023;82:833–955. 10.1016/j.jacc.2023.04.003. 37480922 10.1016/j.jacc.2023.04.003

[18] National Institute for Health and Care Excellence. Chronic kidney disease: assessment and management. NICE Guideline. 2021;203. https://www.nice.org.uk/guidance/ng203 [Accessed 4th March 2025]. 34672500

[19] Wiles K, Chappell L, Clark K, Elman L, Hall M, Lightstone L, et al. The renal association - clinical practice guideline: pregnancy and renal disease. BMC Nephrol. 2019;20:401. 10.1186/s12882-019-1560-2. 10.1186/s12882-019-1560-2 PMC6822421 31672135

[20] Stevens PE, Ahmed SB, Juan Jesus Carrero, Foster B, Francis A, Hall RK, et al. KDIGO 2024 clinical practice guideline for the evaluation and management of chronic kidney disease. Kidney Int. 2024;105:S117–314. 10.1016/j.kint.2023.10.018. 38490803 10.1016/j.kint.2023.10.018

[21] Woodward M. Cardiovascular disease and the female disadvantage. Int J Environ Res Public Health. 2019;16:1165. 10.3390/ijerph16071165. 30939754 10.3390/ijerph16071165 PMC6479531

[22] Sanga V, Seccia TM, Rossi GP. A systematic review of pathophysiology and management of familial hyperaldosteronism type 1 in pregnancy. Endocrine. 2021;74:5–10. 10.1007/s12020-021-02763-5. 34043182 10.1007/s12020-021-02763-5 PMC8440273

[23] Campino C, Trejo P, Carvajal CA, Vecchiola A, Valdivia C, Fuentes CA, et al. Pregnancy normalized familial hyperaldosteronism type I: a novel role for progesterone? J Hum Hypertens. 2015;29:138–9. 10.1038/jhh.2014.49. 24943290 10.1038/jhh.2014.49

[24] Sawami K, Tanaka A, Node K. Appropriate mineralocorticoid receptor antagonism and salt restriction are essential for primary aldosteronism therapy. Hypertens Res. 2023;46:794–6. 10.1038/s41440-022-01155-0. 36609497 10.1038/s41440-022-01155-0

[25] Zhao M, Woodward M, Vaartjes I, Millett ERC, Klipstein‐Grobusch K, Hyun K, et al. Sex differences in cardiovascular medication prescription in primary care: a systematic review and meta‐analysis. J Am Heart Assoc. 2020;9:e014742. 10.1161/jaha.119.014742. 32431190 10.1161/JAHA.119.014742 PMC7429003

[26] Redshaw M, Heikkila K Delivered with care: a national survey of women’s experience of maternity care. 2010. https://www.npeu.ox.ac.uk/assets/downloads/maternity-surveys/reports/Maternity-Survey-Report-2010-Delivered-with-Care.pdf.

[27] Leighton L The giant PANDA study: which blood pressure medication is best for pregnant women with high blood pressure? ISRCTNregistry. 2020. 10.1186/ISRCTN12792616.

[28] Drugs and Lactation Database. Enalapril. Bethesda (MD): National Institute of Child Health and Human Development; 2006. https://www.ncbi.nlm.nih.gov/books/NBK500582/.

[29] Devlin RG, Fleiss PM. Captopril in human blood and breast milk. J Clin Pharmacol. 1981;21:110–3. 10.1002/j.1552-4604.1981.tb01759.x. 7014657 10.1002/j.1552-4604.1981.tb01759.x

[30] Kearney L, Wright P, Fhadil S, Thomas M. Postpartum cardiomyopathy and considerations for breastfeeding. Card Fail Rev. 2018;4:112–8. 10.15420/cfr.2018.21.2. 30206487 10.15420/cfr.2018.21.2 PMC6125711

