# Supplementary material for: RAAS inhibitors in pregnancy, breastfeeding and women of childbearing potential: a review of national and international clinical practice guidelines
Source: J Hum Hypertens. 2025 Mar 5;39(5):315–9. doi: 10.1038/s41371-025-01001-z (PMC12069075; doi:10.1038/s41371-025-01001-z)
Supplement: Supplementary file 1 — Supplementary Tables [file 41371_2025_1001_MOESM1_ESM.pdf]

RAAS inhibitors in pregnancy, breastfeeding and women of childbearing potential: A review of national and international clinical practice guidelines

Supplemental Tables

Caitlin Greenlees and Christian Delles

School of Cardiovascular and Metabolic Health, University of Glasgow, Glasgow, UK

Supplemental Table 1. The national and international clinical guidelines published by reputable health guidance bodies and learned societies in the UK, USA and Europe for the treatment of cardiovascular and renal conditions. Guidelines were reviewed using European and American guidance as standard comparators for the UK NICE guidance.

Guideline				
	**Hypertension**	**Heart Failure**	**Ischaemic Heart Disease**	**Chronic Kidney Disease**
**UK**	2019a NICE. Hypertension in adults: diagnosis and management (5)<br><br>2019b NICE. Hypertension in pregnancy: diagnosis and management (6)	2018 NICE. Chronic heart failure in adults: diagnosis and management (12)	2016 NICE. Stable angina: management (15)	2021 NICE. Chronic kidney disease: assessment and management (18)<br><br>2019 The Renal Association - Clinical Practice Guideline: Pregnancy and Renal Disease (19)
**Europe**	2023 ESH Guidelines for the management of arterial hypertension (7)<br><br>2024 ESH Position Statement on management of hypertensive disorders in pregnancy (11)<br><br>2024 ESC Guidelines for the management of Hypertension (8)<br><br>2018 ESC Guidelines on the management of cardiovascular diseases during pregnancy (10)	2021 ESC Guidelines for the diagnosis and treatment of acute and chronic heart failure (13)<br><br>2018 ESC Guidelines on the management of cardiovascular diseases during pregnancy (10)	2019 ESC Guidelines for the diagnosis and management of chronic coronary syndromes (16)	021 KDIGO Clinical Practice Guideline for the Management of Blood Pressure in Chronic Kidney Disease (20)

**USA**	2017 ACC/AHA/AAPA/ABC/ACPM/AGS/APhA/ASH/ASPC/NMA/PCNA Guideline for the Prevention, Detection, Evaluation, and Management of High Blood Pressure in Adults (9)	2022 AHA/ACC/HFSA Guideline for the management of Heart Failure (14)	2023 AHA/ACC/ACCP/ASPC/NLA/PCNA Guideline for the Management of Patients with Chronic Coronary Disease (17)	

Abbreviations: NICE, National Institute of Health and Care Excellence; ESH, European Society of Hypertension; ESC, European Society of Cardiology; ACC, American College of Cardiology; AHA, American Heart Association; HFSA, Heart Failure Society of America; KDIGO, Kidney Disease: Improving Global Outcomes.

Supplemental Table 2. Published clinical guidelines on the treatment of hypertension with blockers of the renin-angiotensin aldosterone system (RAAS) in pregnancy.

Hypertension			
**Region**	UK	Europe	US
**Guideline by:**	NICE 2019a, 2019b (5,6)	ESC 2018 (10)<br>ESH 2023 (7)<br>ESC 2024 (8)	ACC/AHA/AAPA/ABC/ACPM/AGS/APhA/ASH/ASPC/NMA/PCNA 2017 (9)
**General Statement**	"Stop antihypertensive treatment in women taking ACE inhibitors or ARBs if they become pregnant (preferably within 2 working days of notification of pregnancy) and offer alternatives" (2019b)<br><br>"Note that ACE inhibitors and angiotensin II receptor antagonists should not be used in pregnant or breastfeeding women or women planning pregnancy unless absolutely necessary, in which case the potential risks and benefits should be discussed." (2019a)	"ACE inhibitors and ARBs are teratogenic and contraindicated during pregnancy.[36] Renal or tubular dysplasia, renal failure, oligohydramnios, growth retardation, ossification disorders of the skull, lung hypoplasia, contractures, large joints, anaemia, and intrauterine foetal death have been described. (2018)"<br><br>"RAS inhibitors are not recommended in pregnancy due to adverse foetal and neonatal outcomes. (2024)"<br>"During the first trimester, all RAS[6] blockers, i.e. ACEis, ARBs or direct renin inhibitors should be stopped." (2023)	"ACE inhibitors and ARBs are not approved for use during pregnancy; they are fetotoxic".<br><br>"Women with hypertension who become pregnant should not be treated with ACE inhibitors, ARBs or direct renin inhibitors."
**Other relevant information**	"Advise women who take angiotensin-converting enzyme (ACE) inhibitors or angiotensin II receptor blockers (ARBs):<br>that there is an increased risk of congenital abnormalities if these drugs are taken during pregnancy" (2019b)	"Positive outcomes with ACE inhibitors have been described and pregnancy does not have to be terminated if the patient was exposed to these medications, but should be followed-up closely." (2018)	"Adverse events in the later trimesters have been suggested by observational data and meta-analyses"

	"Offer antihypertensive drug treatment to women of childbearing potential with diagnosed hypertension in line with the recommendations in this guideline. For women considering pregnancy or who are pregnant or breastfeeding, manage hypertension in line with the recommendations on management of pregnancy with chronic hypertension..." (2019a)	"Clinicians should decide on an individual basis whether drug treatment during fetal organogenesis (until week 16) overcomes the risk of fetal drug effects because any drug may be potentially hazardous during the first trimester" (2023)<br><br>"...the use of ACEis in the postpartum period should be reserved for women with cardiorenal comorbidities and is, thus, not recommended in healthy women with hypertensive disorders during puerperium." (2023)	
**Breastfeeding/lactation**	"Note that ACE inhibitors and angiotensin II receptor antagonists should not be used in pregnant or breastfeeding women... unless absolutely necessary in which case the potential risks and benefits should be discussed". (2019a)<br><br>"Where possible, avoid using diuretics or angiotensin receptor blockers to treat hypertension in women in the postnatal period who are breastfeeding or expressing milk...Although ACE inhibitors and angiotensin II receptor antagonists are generally not recommended for use by breastfeeding mothers, they are not absolutely contraindicated." (2019b)	"Antihypertensive drugs taken by the nursing mother are excreted into breast milk, mostly in very low concentrations. Proper information on prescribable drugs in breastfeeding women is important" (2023)<br><br>"ACEis are compatible with breastfeeding and can be used in women with HDP and underlying CVD or CKD. ARBs are not currently recommended in lactating women because of limited safety evidence" (2023)	No mention

		"All BP-lowering drugs are excreted into breast milk. Except for propranolol, atenolol, acebutolol, and nifedipine, most drugs are excreted in very low concentrations in breast milk" (2024)<br><br>Blood pressure-lowering medications that are considered safe with breastfeeding: ACE inhibitors Benazepril, Captopril, Enalapril, Quinapril (2024).	
**Timing of discontinuation of medication**	"Within 2 working days of notification of pregnancy"	"During the first trimester" (2023)	"ACE inhibitors and ARBs are fetotoxic in the second and third trimesters of pregnancy." "Adverse effects in the first trimester of pregnancy may be secondary to hypertension or the medication."
**Replacement of RAAS blockers recommended**	Labetalol, nifedipine, methyldopa (2019b)	Labetalol, alpha-methyldopa and a calcium channel blocker (2023)<br><br>"The BP-lowering drugs of choice are: beta-blockers (most data are available for labetalol, a non-selective beta-blocker that also acts as an alpha-blocker in higher doses; metoprolol and bisoprolol are also considered safe), dihydropyridine CCBs (most data are available for nifedipine, which is	Methyldopa, Nifedipine and/or labetalol

		generally considered first choice, also felodipine, nitrendipine, amlodipine, and isradipine can be used), and methyldopa." (2024)	

Abbreviations: NICE, National Institute of Health and Care Excellence; ESH, European Society of Hypertension; ESC, European Society of Cardiology; ACC, American College of Cardiology; AHA, American Heart Association; AAPA, American Academy of Physician Associates; ABC, Association of Black Cardiologists, ACPM, American College of Preventive Medicine; AGS, American Geriatrics Society; APhA, American Public Health Association; ASH, American Society of Haematology; ASPC, American Society for Preventative Cardiology; NMA, National Medical Association; PCNA, Preventative Cardiovascular Nurses Association; ACE, Angiotensin Converting Enzyme; ARB, Angiotensin Receptor Blocker; RAAS, Renin Angiotensin Aldosterone System; HDP, Hypertensive Disorders of Pregnancy; CVD, Cardiovascular Disease; CKD, Chronic Kidney Disease.

Supplemental Table 3. Published clinical guidelines on the treatment of heart failure with blockers of the renin- angiotensin aldosterone system (RAAS) in pregnancy.

Heart Failure			
**Region**	UK	Europe	US
**Guideline**	2018 NICE (12)	2021 ESC (13)<br>2018 ESC (10)	2023 AHA/ACC/HFSA (14)
**General Statement**	"In women of childbearing potential who have heart failure, contraception and pregnancy should be discussed. If pregnancy is being considered or occurs, specialist advice should be sought."	"ACE-Is, ARBs, ARNI, MRAs, ivabradine, and SGLT2 inhibitors are contraindicated and should be stopped prior to conception with close clinical and echocardiographic monitoring." (2021)	"In women with HF or cardiomyopathy who are planning a pregnancy, ACEi, ARB, ARNi, MRA, SGLT2i, ivabradine and vericigaut should not be administered because of significant risks to fetal harm"
**Other relevant information**	None specified	"Pre-pregnancy management includes the modification of existing HF medications to avoid foetal harm. Angiotensin-converting enzyme (ACE) inhibitors, angiotensin receptor blockers (ARBs), angiotensin receptor neprilysin inhibitors (ARNIs), mineralocorticoid receptor antagonists (MRAs), and ivabradine are contraindicated and should be stopped prior to conception." (2018)<br><br>"Women with advanced HF (LVEF<30%, NYHA class III–IV) in mWHO IV[12] who are pregnant can be referred to a specialist centre for counselling regarding any consideration of termination of pregnancy." (2021)	"ACEi and ARB are associated with second- and third-trimester renal and tubular dysplasia, oligohydramnios, fetal growth restriction, ossification disorders of the skull, lung hypoplasia, contractures, large joints, anemia, and intrauterine fetal death and are, therefore, strictly contraindicated."
**Breastfeeding/lactation**	No mention	No mention	"Postpartum women who breastfeed can start ACEi

			(enalapril or captopril preferred), and metoprolol remains the preferred beta blocker. Within a construct of multidisciplinary shared decision-making, medications that may be appropriate during breastfeeding include ACEi (enalapril or captopril preferred, monitor neonatal weight), beta blockers (metoprolol preferred, monitor neonatal heart rate). Diuretics can suppress lactation, but with neonatal follow-up the use of furosemide may be appropriate”
**Timing of discontinuation of medication**	No mention	“…should be stopped prior to conception” (2018, 2021)<br><br>“If contraindicated drugs have been inadvertently taken during the first trimester, they should be stopped, and the patient monitored closely with maternal echocardiography and foetal ultrasound.” (2018)	“ACEi and ARB are associated with second- and third-trimester renal and tubular dysplasia…”
**Replacement of RAAS blockers recommended**	Diuretics, hydralazine and nitrate, digoxin.	“…beta-blockers should be continued and switched to beta-1-selective blockers.”<br><br>“Beta-1-selective blockers (bisoprolol, metoprolol succinate) and diuretic therapy” (2021)	Beta blocker (most commonly metoprolol)<br>Hydralazine and nitrates<br>Furosemide, digoxin

Abbreviations: NICE, National Institute of Health and Care Excellence; ESC, European Society of Cardiology; AHA/ACC/AFSA, American Heart Association/American College of

Cardiology/Heart Failure Society of America; ACE-I, Angiotensin Converting Enzyme Inhibitor; ARB, Angiotensin Receptor II Blocker; ARNI, Angiotensin Receptor-Neprilysin Inhibitor; MRA, Mineralocorticoid Receptor Antagonist; SGLT2, Sodium-glucose-co-transporter-2; HF, Heart failure; LVEF, Left Ventricular Ejection Fraction; NYHA, New York Heart Association; mWHO, modified World Health Organisation.

Supplemental Table 4. Published clinical guidelines on the treatment of IHD with blockers of the renin-angiotensin aldosterone system (RAAS) in pregnancy.

Ischaemic Heart Disease			
**Region**	UK	Europe	US
**Guideline**	2016 NICE (15)	2019 ESC (16)	2020 AHA/ACC/ACCP/ASPC/NLA/PCNA (17)
**General Statement**	No mention	No mention	"Women with CCD who are contemplating pregnancy or who are pregnant should be risk-stratified and counselled regarding risks of adverse maternal, fetal and obstetric outcomes... and should receive care from a multidisciplinary cardio-obstetric care team beginning before conception and continuing throughout pregnancy, delivery and postpartum to improve maternal and fetal outcomes."<br><br>"Women with CCD who are contemplating pregnancy or who are pregnant should not use ACE inhibitors, ARBs, direct renin inhibitors, angiotensin receptor-neprilysin inhibitors, or aldosterone inhibitors during pregnancy to prevent harm to fetus".
**Other relevant information**	No mention	No mention	"ACE inhibitors, ARBs, direct renin inhibitors, and angiotensin receptor-neprilysin inhibitors can cause renal dysgenesis, oligohydramnios because of fetal oliguria, neonatal anuric renal failure, intrauterine growth retardation, pulmonary hypoplasia, and fetal death, especially when used in the second and third trimester of pregnancy. Benazepril, captopril, and enalapril are safe during lactation. Aldosterone antagonists are contraindicated in pregnancy because of their antiandrogen effects and potential teratogenesis."
**Breastfeeding/lactation**	N/A	N/A	"Benazepril, captopril, and enalapril are safe during lactation. Aldosterone antagonists are contraindicated in pregnancy because of their antiandrogen effects and potential teratogenesis. Aldosterone antagonists are also contraindicated during lactation".
**Timing of discontinuation of medication**	N/A	N/A	"ACE inhibitors, ARBs, direct renin inhibitors, and angiotensin receptor-neprilysin inhibitors can cause renal dysgenesis etc.... especially when used in the second and third trimester of pregnancy."

**Replacements of RAAS blocker recommended**	N/A	N/A	Beta-blockers (most recommended are carvedilol, metoprolol tartrate, metoprolol succinate, nadolol, bisoprolol, propranolol, and timolol)<br><br>Thiazide diuretic therapy or calcium channel blockers added onto beta blocker therapy if outcome does not sufficiently control BP.

Abbreviations: NICE, National Institute of Health and Care Excellence; ESC, European Society of Cardiology; AHA/ACC/ACCP/ASPC/NLA/PCNA, American Heart Association/American College of Cardiology/American College of Clinical Pharmacology, American Society for Preventative Cardiology/National Lipid Association/Preventative Cardiovascular Nurses Association; CCD, Chronic Coronary Disease; ACE, Angiotensin Converting Enzyme; ARB, Angiotensin Receptor II Blocker.

Supplemental Table 5. Published clinical guidelines on the treatment of kidney disease with blockers of the renin-angiotensin aldosterone system (RAAS) during pregnancy.

Chronic Kidney Disease			
**Region**	UK	Europe	US
**Guideline**	2021 NICE (18)<br>2019 The Renal Association (19)	2024 KDIGO (20)	
**General Statement**	No mention (NICE, 2021)<br><br>Antihypertensive agents such as ACE inhibitors, angiotensin receptor blockers and diuretics should be avoided in pregnancy due to potential for fetal harm (The Renal Association, 2019).	"Angiotensin-converting enzyme inhibitor or angiotensin II receptor blocker should be first-line therapy for BP control when albuminuria is present; otherwise dihydropyridine CCB or diuretic can also be considered."<br><br>"Special considerations for CKD care in pregnancy and lactation:<br>• Drug pharmacokinetics and pharmacodynamics<br>• Drug teratogenicity<br>• Risk of CKD progression<br>• Increased risk of pregnancy complications, preterm birth and small for gestational age babies<br>• Fertility"	
**Other relevant information**	No mention (2021)<br><br>We recommend pre-pregnancy counselling for the optimisation of maternal and neonatal outcomes in women with CKD, which may include<br>1. stabilising disease activity in advance of pregnancy on minimised doses of pregnancy-appropriate medications<br>2. minimising risk of exposure to teratogenic medications (The Renal Association, 2019)<br><br>We recommend women with CKD who are taking	"the use of some recommended medications has not been studied in pregnant populations, highlighting the importance of contraceptive counselling in accordance with a person's values and preferences. In other instances, preconception counselling, changing medications to nonteratogenic options and a multidisciplinary approach, is required to optimize the outcomes of a potential pregnancy in the setting of CKD."<br><br>"When prescribing medications to people with CKD who are of child-bearing potential, always review teratogenicity potential and provide regular reproductive and contraceptive counselling in accordance with the values and preferences of the person with CKD.<br><br>When pregnancy is not desired, we note that while the effect of different forms of contraception on GFR is unknown, oral contraceptives are associated with increased BP and hypertension. Nonoral hormonal contraceptives have a less clear impact on BP.	

	angiotensin converting enzyme inhibitors have a plan for discontinuation/conversion guided by the strength of indication for renin-angiotensin blockade and the likelihood of pregnancy confirmation in the first trimester. (The Renal Association, 2019)	
**Breastfeeding/lactation**	No mention (NICE, 2021)<br><br>We recommend women can breastfeed whilst taking prednisolone, hydroxychloroquine, azathioprine, ciclosporin, tacrolimus, enalapril, captopril, amlodipine, nifedipine, labetalol, atenolol and low molecular weight heparin. (The Renal Association, 2019)	"A thorough medication chart review is necessary to replace teratogenic medications before conception, or whenever this is not possible, ensure a strict monitoring plan with cessation of potentially teratogenic medications at conception. A similar approach should be undertaken during lactation recognizing that some medications suitable for use during pregnancy may not be appropriate for lactation, and vice versa. Multidisciplinary care with obstetrics and potentially other subspecialty care is required before conception and throughout pregnancy and lactation."
**Timing of discontinuation of medication**	"In women who have been maintained on these antihypertensive agents whilst waiting to conceive, they should be switched to labetalol or nifedipine (or a suitable alternative) within two days of notification of pregnancy." (The Renal Association, 2019)	Pregnancy may pose a risk of CKD progression for people with established CKD. In addition, some recommended medications to slow or prevent CKD progression are teratogenic (such as ACEi/ARBs or mammalian target of rapamycin inhibitors) and discontinuation during pregnancy should be considered."

**Replacements of RAAS blockers recommended**	No mention (NICE, 2021)<br><br>"...labetalol or nifedipine" (The Renal Association, 2019)	"Some CKD-specific medications should be continued during pregnancies such as hydroxychloroquine, tacrolimus, cyclosporin, eculizumab, prednisone, azathioprine, colchicine, and intravenous immunoglobulin."

Abbreviations: NICE, National Institute of Health and Care Excellence; KDIGO, Kidney Disease: Improving Global Outcomes; ACEi, Angiotensin Converting Enzyme Inhibitor; ARB, Angiotensin Receptor Blocker; CKD, Chronic Kidney Disease; BP, Blood Pressure; GFR, Glomerular Filtration Rate; CCB, Calcium-channel blocker.

References

For references please refer to the main paper.
